# Antibiotic Prophylaxis for Gynecologic Procedures prior to and during the Utilization of Assisted Reproductive Technologies: A Systematic Review

**DOI:** 10.1155/2016/4698314

**Published:** 2016-03-07

**Authors:** Nigel Pereira, Anne P. Hutchinson, Jovana P. Lekovich, Elie Hobeika, Rony T. Elias

**Affiliations:** ^1^The Ronald O. Perelman and Claudia Cohen Center for Reproductive Medicine, Weill Cornell Medical College, New York, NY 10021, USA; ^2^Department of Obstetrics and Gynecology, Weill Cornell Medical College, New York, NY 10021, USA; ^3^Department of Obstetrics and Gynecology, North Shore Long Island Jewish Staten Island University Hospital, Staten Island, NY 10309, USA

## Abstract

The use of assisted reproductive technologies (ART) has increased steadily. There has been a corresponding increase in the number of ART-related procedures such as hysterosalpingography (HSG), saline infusion sonography (SIS), hysteroscopy, laparoscopy, oocyte retrieval, and embryo transfer (ET). While performing these procedures, the abdomen, upper vagina, and endocervix are breached, leading to the possibility of seeding pelvic structures with microorganisms. Antibiotic prophylaxis is therefore important to prevent or treat any procedure-related infections. After careful review of the published literature, it is evident that routine antibiotic prophylaxis is generally not recommended for the majority of ART-related procedures. For transcervical procedures such as HSG, SIS, hysteroscopy, ET, and chromotubation, patients at risk for pelvic infections should be screened and treated prior to the procedure. Patients with a history of pelvic inflammatory disease (PID) or dilated fallopian tubes are at high risk for postprocedural infections and should be given antibiotic prophylaxis during procedures such as HSG, SIS, or chromotubation. Antibiotic prophylaxis is recommended prior to oocyte retrieval in patients with a history of endometriosis, PID, ruptured appendicitis, or multiple prior pelvic surgeries.

## 1. Introduction

The use of assisted reproductive technologies (ART) to overcome infertility has increased steadily in the United States [1]. In 2012, ART contributed to 1.5% of all infants born in the United States [1]. ART procedures consist of several steps over a 2-week period beginning with drug-induced ovarian stimulation, progressing to oocyte retrieval and fertilization with sperm in the laboratory, and ultimately leading to embryo transfer [1]. However, before proceeding with ART, many women may undergo diagnostic procedures to establish the necessity of or potentially modify ART. These procedures, both prior to and during ART, can breach the abdomen, upper vagina, and endocervix, leading to the possibility of seeding the uterus, fallopian tubes, or peritoneal cavity with microorganisms from the skin, vagina, or endocervix [2]. It is therefore important to consider antibiotic prophylaxis to prevent or treat such procedural infections [2]. Recent evidence suggests that alteration of the human microbiome can influence ART outcomes [3]. Thus, in this paper we systematically review the current evidence pertaining to antibiotic prophylaxis for gynecologic procedures prior to and during ART.

## 2. Scope of ART

Ever since the birth of the first US infant in 1981 using ART, the number of ART clinics and procedures performed has increased [1]. An estimated 456 ART clinics in the US performed 157,635 ART procedures in 2012 [1]. These procedures resulted in 51,261 live deliveries and 65,151 infants [1]. In general, ART includes treatments such as in vitro fertilization (IVF), gamete intrafallopian transfer (GIFT), and zygote intrafallopian transfer (ZIFT), with IVF accounting for approximately 99% of all ART procedures [1]. ART, however, does not include treatments such as intrauterine insemination, in which only sperm is handled, or ovulation induction, which involves stimulating oocyte production with oral or injectable drugs [1].

## 3. Microbiology of Gynecologic Infections

Although preprocedural or surgical antisepsis has been associated with an overall decrease in infections, contamination of the procedural site is inevitable [2]. Studies indicate that for most infections following gynecologic procedures or surgery, the source of pathogens is the patient's skin or vagina [2, 4]. The microorganisms usually encountered are gram-positive aerobic cocci (*Staphylococcus*) when the upper and mid-abdominal skin is incised, or anaerobes and gram-negative aerobes when the skin near the groin or perineum is incised [5, 6]. The procedural or surgical site is also exposed to polymicrobial flora of anaerobes and aerobes when the vaginal walls are breached [6–8]. In general, gynecologic laparoscopy does not breach the vaginal walls, and therefore, any infection after such a procedure more commonly results from contaminating skin microorganisms [2, 9]. Transcervical procedures, such as hysterosalpingography (HSG), saline infusion sonography (SIS), and hysteroscopy, and transvaginal procedures such as oocyte retrieval may potentially seed the endometrium, fallopian tubes, or peritoneal cavity with microorganisms from the endocervix or upper vagina [2, 8]. The overall risk of such a procedural infection is rare and mostly occurs in patients with a history of pelvic inflammatory disease (PID) [10].

## 4. Selection of Articles

We searched PubMed, EMBASE, and Google Scholar for all English-language publications between January 1980 and July 2015 with the search terms “antibiotics,” “prophylaxis,” “laparoscopy,” “hysteroscopy,” “hysterosalpingography,” “saline infusion sonography,” and “in vitro fertilization.” The authors independently reviewed the preliminary search results and article titles. Of this initial pool, all authors read relevant abstracts regarding antibiotic prophylaxis for gynecologic procedures, specifically in the context of ART. Given the paucity of prospective randomized control trials (RCTs), all study types, that is, case reports, retrospective cohort studies, prospective case-control studies, and other reviews, were included in the analysis.

**Figure 1:**
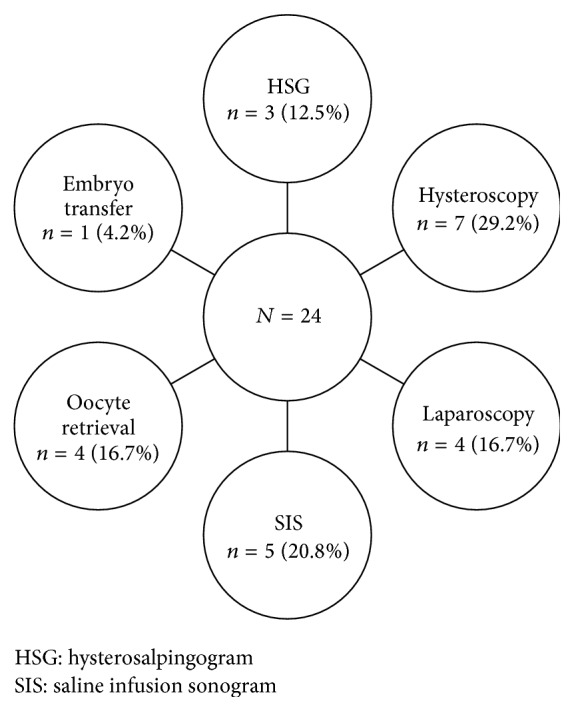


A total of 640 publications were initially identified using the aforementioned search terms. Of these, 24 (3.75%) publications met inclusion criteria. The publications included, by procedure type, were as follows: HSG, 3 (12.5%); hysteroscopy, 7 (29.2%); laparoscopy, 4 (16.7%); SIS, 5 (20.8%); oocyte retrieval, 4 (16.7%); and embryo transfer, 1 (4.2%). The systematic review was performed according to Preferred Reporting Items for Systematic Reviews and Meta-Analysis (PRISMA) guidelines [11].  Figure 1 summarizes the paper's search strategy.

## 5. Prior to ART

Prior to commencing ART, a thorough assessment of the uterus and adnexa is generally performed and often involves bimanual examination and pelvic ultrasonography (US). In addition, previous HSG films should be reviewed for the presence of tubal or uterine abnormalities such as hydrosalpinges and filling defects [12]. To rule out intrauterine lesions such as polyps or leiomyomata, additional evaluation of the uterine cavity can be performed with mid-cycle transvaginal US or SIS [12]. Any abnormal findings noted during mid-cycle US or SIS is generally evaluated with hysteroscopy, which is the gold standard technique for investigating the uterine cavity [13]. Similarly, extrauterine lesions seen in HSG such as hydrosalpinges, peritubal adhesions, or endometriomas should be investigated with laparoscopy [14].

### 5.1. Hysterosalpingography

#### 5.1.1. Brief Technique

HSG allows imaging of the uterine cavity and fallopian tubes with injection of contrast media using fluoroscopic visualization [15]. The endometrial cavity is accessed using aseptic technique after which a small volume (10–30 mL) of contrast agent is administered under intermittent fluoroscopy to visualize the structures to be imaged [15, 16]. Postdrainage images can also be obtained when endometrial pathology is suspected [15, 16]. Most often, HSG is used to evaluate patency of the fallopian tubes and to assess tubal factor infertility.

#### 5.1.2. Antibiotic Prophylaxis

Though uncommon, post-HSG PID complicates roughly 2% of procedures and can have serious implications [17, 18]. A majority of these infections are thought to arise from the ascent of lower genital tract infections with penetration of the cervical barrier. In a retrospective review of 116 women undergoing HSG, the authors reported a 50% incidence of post-HSG PID in patients with culture confirmed* Chlamydia* infection at the time of the procedure [17]. For this reason, it is important to recognize and screen at-risk patients prior to the procedure. HSG should not also be performed in patients thought to have active pelvic infections or purulent cervicitis.

Tubal dilation is also recognized as a risk factor for post-HSG PID. A retrospective review of 278 HSG procedures revealed an 11% risk of post-HSG PID in patients with tubal dilation compared to 1.4% in the general population [18]. Furthermore, no cases of post-HSG PID in patients with normal fallopian tube anatomy were noted. No cases of post-HSG PID were noted in patients with tubal dilation who received doxycycline prophylaxis [2].

Based on these findings, patients at risk for lower genital tract infections including* Chlamydia trachomatis* and* Neisseria gonorrhoeae* should be screened and treated prior to the procedure. In patients with no history of PID, HSG can be performed without antibiotic prophylaxis [2]. If HSG demonstrates dilated fallopian tubes, oral doxycycline (100 mg twice daily for 5 days) should be given to reduce the risk of post-HSG PID [2]. In patients with a history of PID, doxycycline can be administered before the procedure and continued if dilated fallopian tubes are found during HSG [2].

### 5.2. Saline Infusion Sonography

#### 5.2.1. Brief Technique

SIS involves imaging of the endometrial cavity, using real-time US during injection of sterile saline into the uterus [19]. A speculum is placed into the vagina and the cervix is cleaned with an antiseptic solution [20]. A 3.5 French catheter is flushed with sterile normal saline prior to insertion into the endometrial cavity, which reduces the amount of air introduced into the endometrial cavity [20]. Once the catheter is threaded into the endometrial cavity, the speculum is removed and the transvaginal US transducer is placed [20]. Sterile normal saline is slowly instilled into the endometrial cavity under direct sonographic visualization using a 20 mL syringe attached to the catheter [20]. The addition of intrauterine contrast (normal saline) increases the diagnostic accuracy of transvaginal US [14] and allows for the assessment of uterine cavity abnormalities such as polyps, leiomyomata, and adhesions [21–23].

#### 5.2.2. Antibiotic Prophylaxis

Infection after SIS is a rare but acknowledged complication [10, 20]. The low intrauterine pressure with a nonoccluding catheter and the small amount of fluid utilized to evaluate the endometrial cavity generally minimizes the risk of infection [20]. In one retrospective study of 1,153 patients undergoing SIS [24], the authors reported 9 cases of fever within five days of the procedure. While 4 patients had spontaneous resolution of fever in 24 hours, the remaining 5 required antibiotic therapy. Of these, 2 patients experienced infectious complications necessitating surgery for peritonitis. In another study of 81 patients undergoing SIS for postmenopausal bleeding [25], the authors reported 2 cases of post-procedural endometritis requiring antibiotics. Finally, one case report [10] noted bilateral tuboovarian abscesses in a nulligravid woman with no history of sexually transmitted diseases or PID who underwent SIS as part of an infertility evaluation. The administration of prophylactic antibiotics prior to SIS, therefore, is currently not recommended, though it should be based on the patient's individual risk of PID [2]. Some investigators suggest that all patients undergoing SIS for an infertility work-up should receive prophylactic antibiotics [20, 26]. In patients with known hydrosalpinges, prophylactic antibiotics are recommended if the endometrial cavity must be assessed with SIS [19]. SIS should not be performed in women who could be pregnant or in women with active pelvic infections or unexplained pelvic tenderness [19].

### 5.3. Hysteroscopy

#### 5.3.1. Brief Technique

Hysteroscopy is the gold standard for evaluating the endometrial cavity in cases of abnormal uterine bleeding and infertility as well as recurrent pregnancy loss [13, 27]. This procedure can be performed in the office or outpatient surgical setting; the choice generally depends on patient preference, physician skill, and instrument availability [9, 28]. Several diagnostic systems such as small diameter rigid and flexible scopes, as well as operative systems, including monopolar loop cautery [13], bipolar systems [29], microscissors or graspers [13], and hysteroscopic morcellators [30], are currently available, which are utilized based on the complexity of the proposed hysteroscopic surgery. Normal saline, glycine, and carbon dioxide (CO_2_) are the most commonly used distension media for hysteroscopy. After sterile surgical prepping of the vagina, hysteroscopy is carried out by the grasping of the anterior lip of the cervix with a tenaculum, followed by cervical dilation to the required diameter of the hysteroscope, insertion of hysteroscope, and distention of the uterine cavity with the distension media [13]. Other atraumatic hysteroscopic techniques such as the vaginoscopic approach have also been introduced [27, 31, 32].

#### 5.3.2. Antibiotic Prophylaxis

As with any surgical intervention, hysteroscopy carries risk of infection, but, given the abundance of lower genital tract flora, this risk is small, that is, ranging between 0.18 and 0.55% [2]. In one retrospective cohort study of 200 patients undergoing operative hysteroscopy without antibiotic prophylaxis [33], the investigators reported 3 (1.5%) cases of severe pelvic infection. All patients with postsurgical infections had a prior history of PID. In another large retrospective study of 2,116 operative hysteroscopies, the authors reported 30 (1.42%) postoperative infections [34]. Of these infections, 18 (0.85%) were cases of endometritis, while the remaining were urinary tract infections. Regarding endometritis, sixteen cases were early-onset and vaginal cultures in this group grew* Streptococcus* D and* Staphylococcus aureus* (2 cases each). Six patients did not have vaginal cultures performed and no pathogens were isolated in the remaining 6 patients. In the 2 cases of late-onset endometritis, vaginal cultures showed* Streptococcus* D in one case and polymicrobial flora in the other case. All patients with endometritis were treated with antibiotics.

A prospective study has investigated the efficacy of amoxicillin and clavulanate antibiotic prophylaxis in preventing bacteremia during hysteroscopy [35]. In this study of 166 patients, the investigators randomized 55 patients to receive 1.2 grams of intravenous antibiotics and 61 patients to receive no antibiotics prior to hysteroscopy. Blood cultures collected at the end of the procedure showed an increased incidence of bacteremia in the nonantibiotic group (16%) compared to the antibiotic group (2%). Despite these differences in bacteremia, no difference in the incidence of postoperative infection was noted. The investigators, therefore, hypothesized that most of the microorganisms isolated may have resulted from contamination.

In their randomized trial of 631 women undergoing diagnostic hysteroscopy with or without antibiotic prophylaxis, Kasius et al. [36] found no significant difference in incidence of postoperative infection. Similarly, in a randomized control trial of 364 women by Gregoriou et al. [37], the investigators found no significant difference in the incidence of postoperative infections between women who received antibiotic prophylaxis and those who did not. In a multicenter, double-blinded, randomized, placebo-controlled study of 1046 patients undergoing hysteroscopy, Nappi et al. [38] assessed the protective effect of prophylactic antibiotic administration. Patients were randomized to receive 1 gram of intramuscular cefazolin (*n* = 523) or placebo (*n* = 523) preoperatively. The investigators found an overall infection rate of 1.15% (*n* = 12) with no statistical difference between the study or control groups. Based on these published studies, most professional societies recommend against routine antibiotic prophylaxis for hysteroscopy in the general patient population [2, 13].

### 5.4. Laparoscopy

#### 5.4.1. Brief Technique

In current clinical practice, a laparoscopy is usually performed for the definitive diagnosis of endometriosis before pursuing ART [14]. In many cases, laparoscopy with excision of hydrosalpinges is performed to improve ART treatment success [39, 40]. Diagnostic laparoscopy generally begins with placing the patient in dorsal lithotomy position, surgical prepping, and draping, followed by the insertion of a Foley catheter. If required, a nasogastric tube and uterine manipulator may also be placed. Open entry of the fascia at the level of the umbilicus or closed entry using a Veress needle in the umbilicus or left upper quadrant of the abdomen is performed next. Following insufflation of the peritoneal cavity with CO_2_, three 5 mm trocars are placed in the umbilicus, right and left lower quadrants of the abdomen. A 0° or 30° 5 mm laparoscope is placed through the umbilical port. Accessory instruments through the other 5 mm ports are used as needed. Chromotubation of the fallopian tubes at the time of diagnostic laparoscopy may also be carried out [2].

#### 5.4.2. Antibiotic Prophylaxis

Over the past decade, laparoscopy has grown in popularity among gynecologists due to its improved cosmesis and postoperative recovery times when compared to laparotomy. In comparison to conventional laparotomy for benign gynecologic conditions, laparoscopic surgery carries a low postoperative infection rate [41]. Laparoscopic procedures can be divided into clean or clean-contaminated (involving entry into the uterine cavity or vagina) procedures [2, 4]. Though most evidence suggests that antibiotic prophylaxis is not required in clean laparoscopic procedures [2, 4], a recent survey of practice patterns in gynecologic surgery found that 54% of practitioners continue to use routine antibiotic prophylaxis for these procedures [42].

In 2005, a placebo-controlled, randomized trial of 450 women undergoing laparoscopy was randomly assigned to receive a single dose of a first generation cephalosporin prior to surgery or placebo [43]. No significant difference was found in either the incidence of postoperative infection or in the mean hospital stay, suggesting no benefit of routine antibiotic prophylaxis in laparoscopy. In support of these findings, a 2010 cohort study including 300 women found no significant difference in the postoperative infection rate between women receiving 2 grams cefazolin prior to surgery and those receiving no antibiotic prophylaxis [44]. Recently, a placebo-controlled, randomized trial similarly found that, in a group of 218 patients undergoing laparoscopy for uncomplicated gynecologic conditions, there was no significant reduction in postoperative infection [45]. Based on the results of the aforementioned studies, antibiotic prophylaxis is recommended against in clean laparoscopic procedures [2, 4]. Like HSG, if abnormal or dilated fallopian tubes are noted during chromotubation, postoperative prophylaxis with doxycycline should be considered [2].

## 6. During ART

### 6.1. Oocyte Retrieval

#### 6.1.1. Brief Technique

In the early days of ART, most oocyte retrievals were performed laparoscopically with general anesthesia [45]. However, today, transvaginal US-guided oocyte retrieval under sedation or local anesthesia has become the current standard of care [46, 47]. Though conscious sedation is the most common form of anesthesia for oocyte retrievals, local, spinal, epidural, or general anesthesia is sometimes utilized [12]. Oocyte retrievals are performed with the patient in dorsal lithotomy position after prepping the vagina and perineum with povidone-iodine or hexachlorophene solution and copious irrigation with sterile saline solution [12]. A high-frequency transvaginal US transducer laden with a needle sheath is used to visualize the ovaries. A 30 cm, 16 G, single-lumen or double-lumen aspiration needle is used to puncture the ovarian follicles using the needle sheath as a guide [12]. A constant pressure of 80–100 mm Hg assists in the collection follicular fluid [12].

#### 6.1.2. Antibiotic Prophylaxis

The overall incidence of infection after an oocyte retrieval is estimated to be about 0.4% [48, 49]. In most instances, pelvic infections after oocyte retrievals have been noted in patients who have endometriosis, likely due to presence of pelvic adhesions [50, 51]. At our center, a 2 gram dose of intravenous cefoxitin is routinely administered prior to oocyte retrieval as prophylaxis in all patients with a history of endometriosis, PID, ruptured appendicitis, or multiple prior pelvic surgical procedures [12]. All oocyte donors also receive the aforementioned antibiotic prophylaxis prior to oocyte retrieval [12]. Prospective trials are currently lacking to validate the generalizability of this antibiotic regimen.

### 6.2. Embryo Transfer

#### 6.2.1. Brief Technique

The embryo transfer (ET) procedure is perhaps the most critical step of ART [12]. The main objective of ET is to transfer a good-quality cleavage stage (day 3) embryo or blastocyst (day 5/6) to the uterine cavity in an atraumatic fashion so as to maximize the chances of implantation [12]. Although various commercially available catheters have been utilized in different ART clinics to perform ET, soft ET catheters are generally preferred [12]. When performing ETs, the patient is placed in dorsal lithotomy position and an appropriate-sized speculum is placed in the vagina [12]. The cervix is cleaned with culture media and excess cervical mucus is removed atraumatically to reduce any ascending bacterial contamination [12]. The soft transfer catheter is loaded with the embryo(s) and then inserted into the uterine cavity through the cervical canal. ET can sometimes be aided with US guidance, particularly in patients with previous cesarean deliveries or uterine leiomyomata. Following placement of the embryo(s), the catheter is slowly withdrawn from the uterus [12].

#### 6.2.2. Antibiotic Prophylaxis

In addition to technical aspects, ET can be affected by the genital tract microbial milieu [52]. Clinical pelvic infection is rare after ET [53]; however, there is some evidence that increased endocervical microbial colonization at the time of ET is associated with lower pregnancy rates [54–56]. One systematic review to date has evaluated the effectiveness and safety of prophylactic antibiotic administration prior to ET during ART cycles [52]. Although the authors identified 4 studies, only 1 study [57] was included in the final analysis. This study was a prospective trial of 350 patients randomized to receive a combination of oral amoxicillin (500 mg) and clavulanate (125 mg) on the day before and the day of ET versus no antibiotics. Although antibiotics reduced ET catheter contamination rates, there was no difference in rate of post-ET infections or clinical pregnancy. Thus, these do not support the routine use of antibiotics at ET.

## 7. Summary of Current Evidence

Table 1 summarizes the antibiotic prophylaxis regimens for gynecologic procedures prior to and during ART based on published evidence [2, 3, 8, 49, 52].

## 8. Conclusions

It is important for clinicians to appreciate when antibiotic prophylaxis is indicated and when it is inappropriate. Appropriate antibiotic usage will reduce infectious postoperative complications and minimize the development of antibiotic-resistant organisms. While routine antibiotic prophylaxis is not recommended for the low-risk gynecologic procedures associated with ART, the assessment of individual risk factors remains crucial. For transcervical procedures such as HSG, SIS, hysteroscopy, embryo transfer, and chromotubation, patients at risk for pelvic infections should be screened and treated prior to the procedure. Patients with a history of PID or dilated fallopian tubes are at high risk for postprocedural infections and careful consideration should be given to the risks and benefits of antibiotic prophylaxis in these patients. For transvaginal procedures like oocyte retrieval, antibiotic prophylaxis is recommended in patients with a history of endometriosis, PID, ruptured appendicitis, or multiple prior pelvic surgeries.

## Figures and Tables

**Table 1:** Summary of evidence pertaining to antibiotic prophylaxis regimens for gynecologic procedures prior to and during ART.

Procedure	Guidelines for antibiotic prophylaxis
Hysterosalpingography (HSG)	No prophylactic antibiotics in patients without history of pelvic inflammatory disease (PID) If HSG demonstrats dilated fallopian tubes, oral doxycycline 100 mg should be given twice daily for 5 days In patients with a history of pelvic infection, doxycycline should be given prior to procedure and continued if dilated fallopian tubes visualized

Saline infusion sonography	Routine administration of prophylactic antibiotics is currently not recommended, though it should be based on the patient's individual risk of PID In patients with known hydrosalpinges, prophylactic antibiotics are recommended if the endometrial cavity must be assessed

Hysteroscopy	Routine antibiotic prophylaxis is not recommended

Laparoscopy	Antibiotic prophylaxis is not recommended for laparoscopic procedures that involve no direct access from the abdominal cavity to the uterine cavity or vagina If abnormal or dilated fallopian tubes are noted during chromotubation, postoperative prophylaxis with doxycycline should be considered

Oocyte retrieval	Antibiotic prophylaxis (2 grams intravenous cefoxitin) is suggested in patients with a history of endometriosis, PID, ruptured appendicitis, or multiple prior pelvic surgical procedures Antibiotic prophylaxis (2 grams intravenous cefoxitin) is suggested in oocyte donors

Embryo transfer	Routine antibiotic prophylaxis is not recommended
